# Diversity of endogenous avian leukosis virus subgroup E (ALVE) insertions in indigenous chickens

**DOI:** 10.1186/s12711-020-00548-4

**Published:** 2020-06-01

**Authors:** Andrew S. Mason, Katarzyna Miedzinska, Adebabay Kebede, Oladeji Bamidele, Ahmed S. Al-Jumaili, Tadelle Dessie, Olivier Hanotte, Jacqueline Smith

**Affiliations:** 1 The University of York, York, YO10 5DD UK; 2 The Roslin Institute and Royal (Dick) School of Veterinary Studies, The University of Edinburgh, Easter Bush, Midlothian, EH25 9RG UK; 3 LiveGene–CTLGH, International Livestock Research Institute (ILRI), Addis Ababa, Ethiopia; 4 Addis Ababa University, Addis Ababa, Ethiopia; 5 African Chicken Genetic Gains, Department of Animal Sciences, Obafemi Awolowo, Ile Ife, Osun Nigeria; 6 School of Life Sciences, The University of Nottingham, University Park, Nottingham, NG7 2RD UK; 7 University of Anbar, Ramadi, Anbar Iraq

## Abstract

**Background:**

Avian leukosis virus subgroup E (ALVE) insertions are endogenous retroviruses (ERV) that are restricted to the domestic chicken and its wild progenitor. In commercial chickens, ALVE are known to have a detrimental effect on productivity and provide a source for recombination with exogenous retroviruses. The wider diversity of ALVE in non-commercial chickens and the role of these elements in ERV-derived immunity (EDI) are yet to be investigated.

**Results:**

In total, 974 different ALVE were identified from 407 chickens sampled from village populations in Ethiopia, Iraq, and Nigeria, using the recently developed obsERVer bioinformatics identification pipeline. Eighty-eight percent of all identified ALVE were novel, bringing the known number of ALVE integrations to more than 1300 across all analysed chickens. ALVE content was highly lineage-specific and populations generally exhibited a large diversity of ALVE at low frequencies, which is typical for ERV involved in EDI. A significantly larger number of ALVE was found within or near coding regions than expected by chance, although a relative depletion of ALVE was observed within coding regions, which likely reflects selection against deleterious integrations. These effects were less pronounced than in previous analyses of chickens from commercial lines.

**Conclusions:**

Identification of more than 850 novel ALVE has trebled the known diversity of these retroviral elements. This work provides the basis for future studies to fully quantify the role of ALVE in immunity against exogenous ALV, and development of programmes to improve the productivity and welfare of chickens in developing economies.

## Background

**Fig. 1:**
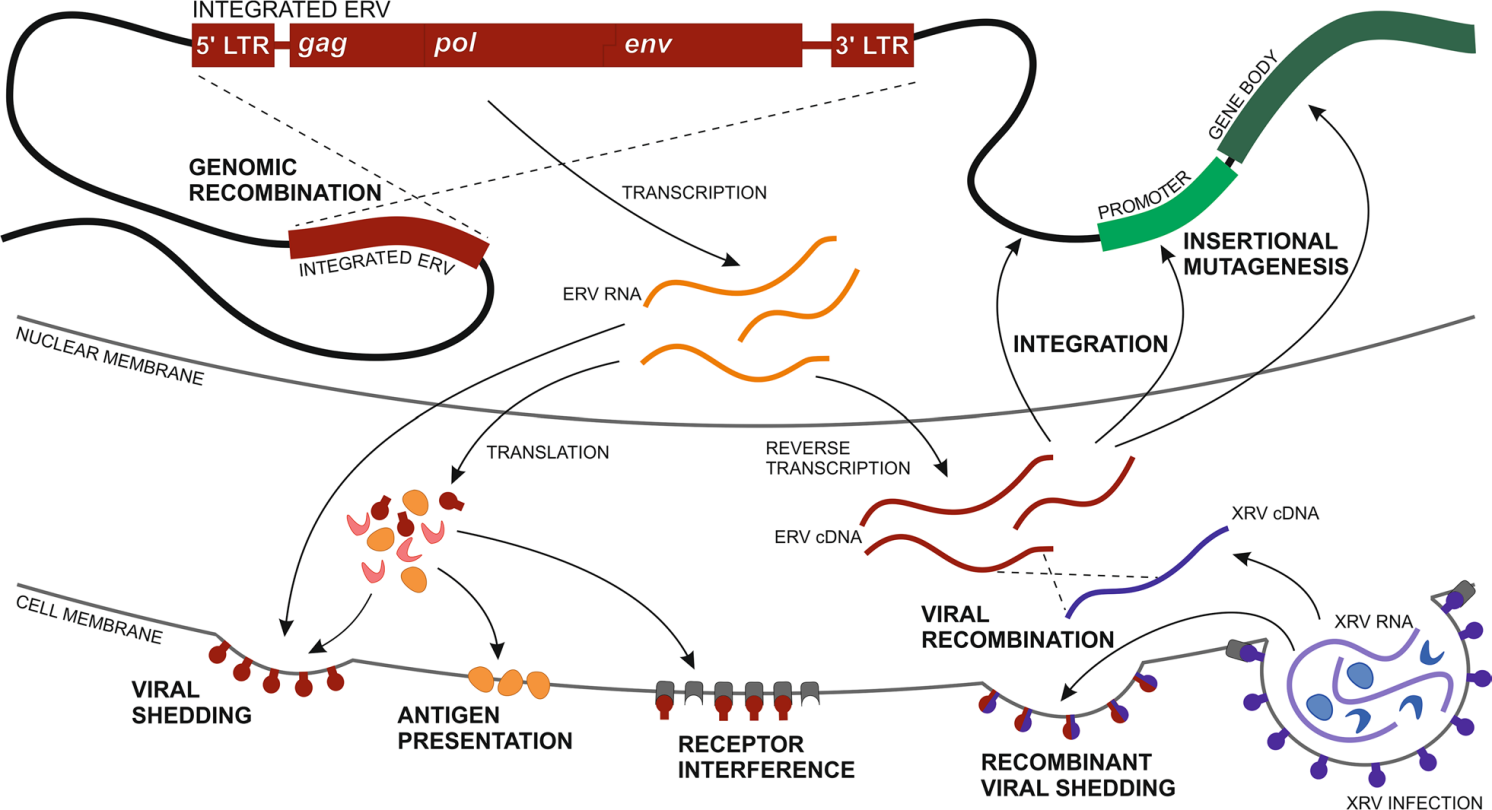
The diverse impacts of endogenous retroviruses. Intact endogenous retroviruses (ERV) share a conserved archetypal structure of retroviral proteins (gag, pol and env) enclosed by two long terminal repeats (LTR) which are identical at the point of integration in the host genome. ERV integration site largely determines its immediate impact on the host, as integration within or near genes may modulate host gene expression and facilitate continued ERV expression of retroviral gene products or intact virions, which can elicit persistent physiological stress on the host. As ERV copy number increases in the genome, ERV recombination facilitates intra- and interchromosomal rearrangements and acts as recipient sequence for recombination with related exogenous retroviruses (XRV)

Retroviruses exhibit persistent yet highly changeable stress on their vertebrate hosts. Insertional mutagenesis can elicit a wide range of phenotypic effects and the rapidly evolving retroviral genome presents a constant immune challenge [1–3]. Furthermore, if a retrovirus integrates within the genome of the germline, these “endogenous” retroviruses (ERV) are inherited vertically, and can continue to affect the host organism over large evolutionary timescales. Thus, ERV provide a genomic record of ancestral retroviral infections, and may elicit novel physiological stress by continuing to retrotranspose, produce retroviral proteins, and recombine, both across the genome and with exogenous retroviruses (Fig. 1) [3–9]. However, the effects of ERV are diverse, with some conferring resistance to new exogenous retroviral infections by three main strategies: receptor interference; inhibition of the retroviral lifecycle (uncoating, reassembly and nuclear localisation); and marking of retroviral RNA for degradation through formation of double stranded RNA [10–15]. Combined, these processes induce varying extents of ERV-derived immunity (EDI) in the host organism. EDI has been observed across vertebrates but elicits a largely transient response over evolutionary timescales, as ERV are retained while they confer a selective advantage and are then strongly selected against when that advantage is lost [2, 15–17].

In chickens (*Gallus gallus*), where ERV represent about 3% of the genome [18, 19], the only retrovirus with recurrent exogenous and endogenous activity is the avian leukosis virus (ALV) [20, 21]. ALV can infect all galliform birds, however subgroup E (ALVE) integrations are found only in the domestic chicken and its wild progenitor, the red junglefowl (RJF) [22]. ALVE have long been known to facilitate EDI [23, 24], but they have been primarily studied in commercial layer lines, where any selective benefit is masked by their typically negative association with productivity traits, and the absence of ALV infection in commercial stock [25–28].

A set of recent studies [29, 30] has begun to scratch the surface of true ALVE diversity within chickens, but primarily in commercial lines. A much broader characterisation of ALVE in non-commercial chickens is required to quantify the extent to which ALVE derive immunity to exogenous ALV. Furthermore, characterising the abundance of ALVE with known negative effects on productivity, or identifying novel ALVE that elicit positive effects on productivity or environmental adaptation, may lead to improvement of chicken meat and egg production in non-commercial settings. In this study, ALVE were identified in the genomes of 407 village chickens from Ethiopia, Iraq, and Nigeria to characterise ALVE diversity more comprehensively, and to assess the likely evolutionary and immunological significance of ALVE in a non-commercial setting.

## Methods

### Animals and sequencing data

Whole-genome (re)sequencing (WGS) data were analysed from 407 chickens (see Additional file 1) as part of the Centre for Tropical Livestock Genetics and Health (CTLGH) Poultry Genetics programme (http://www.ctlgh.org). Chickens were sourced from Ethiopia (n = 260 from 25 populations), Iraq (n = 27 from 3 populations) and Nigeria (n = 120 from 14 populations). The sampled regions and numbers of sequenced individuals are summarised in Additional file 2: Table S1. Geographical data (altitude, vegetation cover, soil type) were available for each sampled region and phenotypic (weight, age, sex, relatedness, feather colour) and epidemiological (previous illnesses and treatment) data were recorded for individual chickens but were incomplete across all populations in each country, particularly in Nigeria and Iraq. All sequencing reads (Illumina 150 bp paired-end) were quality-checked and trimmed where necessary [31–33].

### ALVE identification

ALVE integrations were identified in the WGS data using the bioinformatics pipeline obsERVer, which has been used to identify ALVE in a wide range of chicken datasets [30]. Briefly, obsERVer maps WGS reads to an “ALVE pseudochromosome” that consists of 11 publicly available GenBank ALV sequences [30], extracts mapped reads and their read mates, and aligns these to the Gallus_gallus-5.0 chicken reference genome (Galgal5; GenBank: GCF_000002315.4), removing reads that map to assembled alpharetroviral integrations. A mapping quality greater than 20 was required for the pseudochromosome and reference genome alignments, and reads with secondary alignments within Galgal5 were removed after filtering assembled alpharetroviral integrations. Putative ALVE integrations were annotated by known ALVE sites and manually validated after inspection using the Integrative Genomics Viewer (IGV) v2.4.3 software [34].

### Validation of identified ALVE integrations

Previous validation of obsERVer-detected sites by PCR-based assays [30] showed high sensitivity with a false detection rate (FDR) of 0%. However, given the diversity of the chicken populations in this study, and the high proportion of novel and lineage-specific ALVE, we performed additional validation. Twenty putative ALVE integration sites were selected at random from all novel ALVE integrations detected in this study to act as the validation set. For each of the 20 ALVE in the validation set, six sequenced individuals were chosen to represent the bioinformatically-predicted homozygous wildtype, homozygous integration, and heterozygous integration genotypes, where possible. Some individuals were used to validate multiple integrations (see Additional file 2: Table S2). Specific PCR assays were developed for each integration site using Primer3 v4.1.0 [35] (see Additional file 2: Table S3). PCR reactions were conducted using the Roche FastStart™ Taq DNA polymerase kit (Roche 04738357001) in 10 μl reaction volumes with equal concentrations of primers. PCR began with an activation step at 95 °C for 4 min, followed by 35 cycles of 30 s denaturing at 95 °C, 30 s annealing at 60 °C, and 45 s elongation at 72 °C, with a final extension step at 72 °C for 7 min. PCR products were detected on the Agilent 4200 TapeStation System using High Sensitivity D1000 ScreenTape (Agilent 5067–5584), following the manufacturer’s instructions.

### ALVE distribution analysis

All bioinformatically identified ALVE were combined to identify patterns in their genomic distribution. A dataset of an equal number of randomly generated insertions across Galgal5 was used to identify any skews and biases in distribution, with the simulation repeated one million times. This simulated dataset was compared with the observed GC distribution for the target site duplication and windows of 100 bp, 1 kb, 10 kb, and 100 kb centred on the integration, and the distribution of ALVE relative to coding regions (Ensembl v87). Significant deviations across observed and simulated distributions were assessed with the two-sample Kolmogorov–Smirnov test, and between individual groups using a binomial test. Pearson correlations were derived between the ALVE distribution and log_10_ transformed values for assembled chromosome length, gene density, and chromosome-level recombination rate (converted to Galgal5 from [36]). Significant deviation from the simulated data was assessed using the Fisher *z*-transformation.

### Direct ALVE genotyping and clustering

Reads from each of the 407 datasets were mapped to Galgal5 using the BWA-mem v0.7.10 software [37], and the alignment maps were used to genotype each ALVE insertion. All identified ALVE were used for genotyping and all genotyping results correlated exactly with sites identified by obsERVer for each bird. A binary presence/absence matrix for each ALVE within each individual was generated using 0 for the homozygous wild type and 1 for individuals that were homozygous at the ALVE insertion. This high dimension data was visualised using both t-distributed stochastic neighbour embedding (t-SNE) [38] and hierarchical clustering with Jaccard distances, excluding the ALVE that were found in one individual only. Genotypes were correlated with available geographic, phenotypic and epidemiological data for each bird.

## Results and discussion

### Distribution of ALVE across populations

ALVE were detected from the WGS data of 407 individual chickens that were sampled from village populations in Ethiopia, Iraq, and Nigeria (see Additional file 2: Table S1). In total, 974 different ALVE were identified, with 6053 occurrences and an average of 14.9 ALVE per chicken. The number of ALVE per chicken was highly variable, ranging from six (comparable to levels in commercial brown egg layers [30]) to a maximum of 33. All populations across the three sampled countries exhibited a similar level of diversity. We identified 857 novel ALVE (88.0%), which brings the known diversity of ALVE to over 1300 different integration sites [29, 30]. PCR assays were developed for 20 randomly selected novel ALVE integration sites (see Additional file 2: Table S2) to assess the obsERVer FDR, which was previously shown to be 0% in a commercial chicken dataset [30]. All selected integration sites were successfully validated by PCR (see Additional file 3: Figure S1), which confirmed an obsERVer FDR of 0% and that obsERVer is highly specific for the detection of ERV from WGS data.

Many of the previous ALVE detected in commercial chickens were also found in these indigenous chicken populations [29, 30, 39]. However, it is unclear whether these represent the natural origins of these ALVE, or result from later introduction of Western commercial breeds. Among the identified ALVE, the commercially relevant ALVE21 was the most common. ALVE21 is a replication competent provirus that is associated with the sex-linked slow-feathering *K* locus [40–42], and was present in 75% of all individuals and in all but one of the analysed populations (Dara Kumato, Ethiopia). ALVE1, ALVE3, ALVE15, ALVEB5 and ALVE-TYR were commonly found in all regions, as were ALVE_ros003, 010, 011, 159 and 276, which were previously identified in commercial layers and broilers, and a range of sites that were previously identified in two Ethiopian populations [30].

In total, 393 ALVE (40.3%) were identified only in one individual and, within each population, 40 to 80% of the sites were detected in one bird only. This high diversity of low-frequency ALVE is typical of ERV-derived immunity (EDI), for which ERV are transiently beneficial to the host, since they provide resistance to new retroviral infections by receptor interference [15–17]. This has long been observed with the envelope protein of ALVE [23, 24, 43, 44], and with beta- and gammaretroviral ERV in mammalian species [10–12].

We found no ALVE that were fixed within a population, with the typical maximum ALVE population frequency ranging from 0.45 to 0.60 and a typical average frequency of 0.10 across all ALVE in a population. It is, however, possible that ALVE21 was fixed in seven of the analysed populations (see Additional file 1), in spite of the predominance of heterozygotes, caused by its presence in only one segment of the *K* locus tandem repeat [30, 40]. Some of the homozygous ALVE21 genotypes may result from a reversion event at the *K* locus [45], as was recently observed in commercial White Plymouth Rock layers [30, 46].

**Fig. 2:**
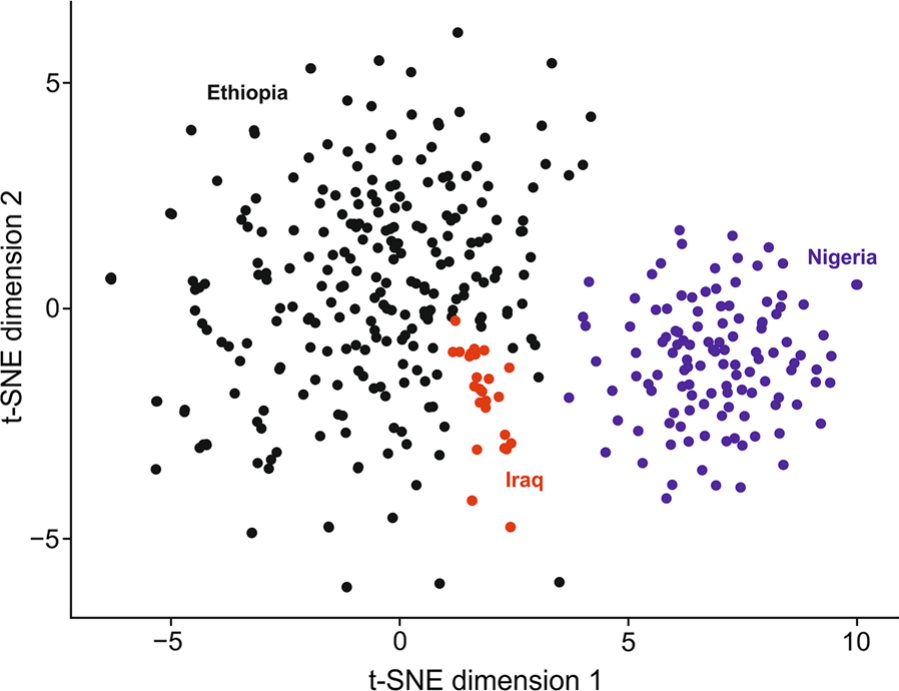
t-SNE visualisation of the ALVE-resolved population structure of the sampled chicken populations. Dimension reduction was performed on a binary matrix of ALVE shared between at least two individuals (n = 581). Samples from each country are coloured black for Ethiopia, red for Iraq and blue for Nigeria. t-SNE was derived using sci-kit learn with Python 3.7 with a learning rate of 15, perplexity of 65, and a maximum of 10,000 iterations to ensure stability

No significant associations with phenotypic or epidemiological data were identified for any ALVE or group of ALVE, although the metadata was incomplete. However, ALVE genotypes were sufficient to reconstitute the geographical distribution of the sampled chickens at the national level (Fig. 2). The Iraqi samples were closely associated with those from the edge of the Ethiopian cluster, but the Nigerian populations were completely distinct, likely reflecting the relative geographical positions of the three countries. However, in most cases, we were not able to unambiguously resolve the population or regional level within each country based on ALVE genotypes alone (see Additional file 4: Figure S2). The relatively poor intra-national and predictable international resolution likely reflects the prevalence of trade within, rather than between, countries. It is possible that the resolution provided by ALVE genotypes is not sufficient to distinguish between closely related populations within a country, but that resolution could be improved by the incorporation of genetic variants that exist in larger numbers, such as single nucleotide variants (SNVs).

### Distribution of ALVE across the genome

**Fig. 3:**
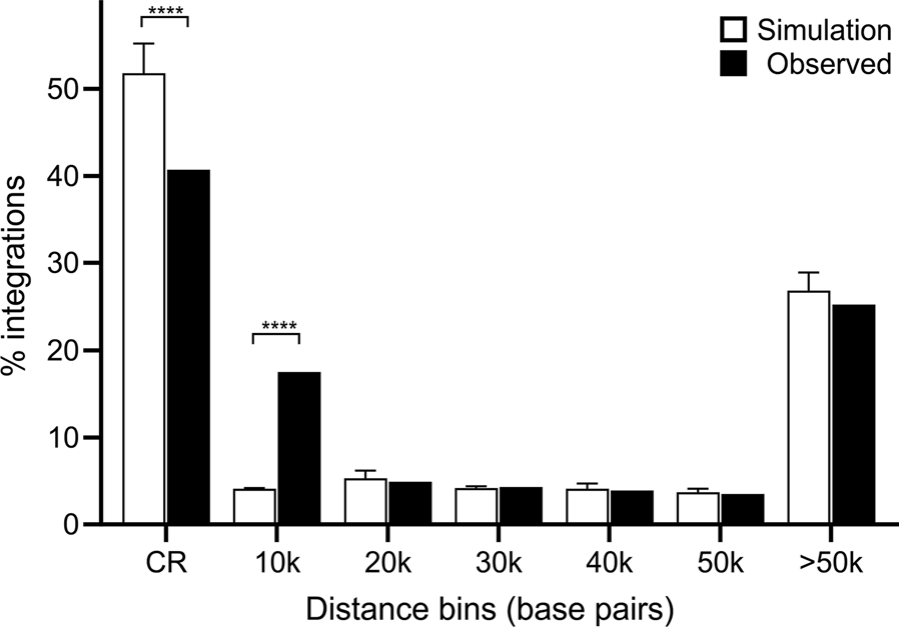
ALVE distribution relative to coding features and randomly simulated integrations. Observed values represent all ALVE identified in this study (n = 974). Simulated values show the mean and standard deviation of one million randomly simulated redistributions of 974 integrations across the Galgal5 assembly. There was a significant depletion (P = 1.74 × 10^−14^) of integrations within coding regions (CR) and significant enrichment (P = 7.16 × 10^−19^) of integrations within 10 kb of CR. All other distance bins had non-significant differences. Specific values are reported in Additional file 2: Table S4

Integration of exogenous ALV occurs preferentially in open chromatin, particularly near protein-coding genes [47–49]. Although ALVE may exhibit the same biological preference, selection acts to remove deleterious endogenous elements over time. Accordingly, a recent analysis of ALVE in a dataset dominated by commercial chickens showed a significant depletion of ALVE within coding regions (26.7% compared to 51.8% of modelled random integrations) but an eightfold enrichment of integrations within 10 kb of a protein-coding gene (32.9% compared to 4.1% of modelled random integrations) [30]. Here, we observed a similar, but less extreme pattern of ALVE distribution (Fig. 3 and Additional file 2 Table S4), with 40.7% of ALVE located within coding regions (depletion; P = 1.74 × 10^−14^) and 17.5% within 10 kb of protein-coding genes (enrichment; P = 7.16 × 10^−19^). These results likely reflect the much less intense selection of these village chickens compared to commercial chickens. Even with the apparent selection against integrations in coding regions, overall these data still indicate a significant enrichment of ALVE within or near protein coding genes (P = 0.03). This enrichment is also evidenced by the significantly elevated GC content of the ALVE target site duplications (KS = 0.38; P = 7.22 × 10^−50^), although this effect was not observed for any other window size that was used for GC content calculation. Taken together, our results indicate that the distribution of ALVE is certainly not random. Given the structure of the chicken genome, ALVE density was highly correlated with chromosome length (r = 0.72; P = 3.03 × 10^−5^), but significantly less than expected with random integration (r = 0.97; z = 26.56; P = 2.51 × 10^−154^). ALVE density had weaker, negative correlations with recombination rate and gene density. However, the variance in both these measures is largely explained by chromosome length (r^2^ = 0.86 and r^2^ = 0.83, respectively).

### Integrations of ALVE within exons

Only six of the 396 ALVE (1.5%) found within coding regions were located in exons, which is significantly less than the 4.9% expected under random integration (P = 6.36 × 10^−4^; see Additional file 2: Table S5). Two of these (ALVE_ros845 and ALVE_ros1003) were found in exon 4 of the *pannexin1* (*PANX1*) gene, a gap junction family member that is expressed throughout the central nervous system. Both of these ALVE were identified in chickens from the Ethiopian Dibate region, and it is likely that the two sites have a shared history: they appear to be only 7 bp apart, but ALVE_ros845 is associated with a genomic deletion that is likely to have a greater impact on *PANX1* function. Two of the other exonic integrations were identified only in single individuals: ALVE_ros529 in the second exon of the *cyclin dependent kinase 15* (*CDK15*) gene, which is known to regulate anti-apoptosis [50], and ALVE_ros586 in exon 4 of the *IQ and ubiquitin*-*like domain*-*containing protein* (*IQUB*) gene, which is involved in the regulation of cilia and the hedgehog signalling pathway [51]. Interestingly, both ALVE_ros569 and ALVE_ros638 were identified in individuals from different regions, with the former found only in one individual from Nigeria and one from Ethiopia. ALVE_ros569 is in exon 2 of the *threonine synthase*-*like 2* (*THNSL2*) gene and may influence the ability of the bird to elicit an appropriate inflammatory response [52, 53], which is particularly relevant during persistent, ALVE-induced viremia. ALVE_ros638 may also influence response to viral load and regulation of anti-apoptosis due to its integration in exon 8 of the *multidrug resistance*-*associated protein 6* (*ABCC6*, encoding the MRP6 protein) gene, however the distinct roles of *MRP6* and a closely related truncated duplicate (*URG7*) are yet to be fully resolved [54].

It is also possible that integration of ALVE in these particular genes reflect a degree of selection (particularly with some sites in distant populations), as each affected gene is part of a large network with multiple redundancies. It would be of great interest to study the specific effects of exonic ALVE insertions, and to identify whether such integrations are tolerated by the host or actively selected against.

## Conclusions

This study is the first step towards characterization of the diversity of ALVE that are present in non-commercial chickens. We identified 857 novel ALVE from a survey of more than 400 indigenous chickens from Ethiopia, Iraq, and Nigeria and observed a diverse pool of low frequency ALVE integrations. Further work is needed to characterise the evolutionary and immunological roles of ALVE within these populations, but our observations are typical of a role in ERV-derived immunity. Six novel ALVE were identified within genes which warrant further investigation to determine their specific effects on the host. Identification of ALVE with detrimental effects on productivity may help guide local breeding programmes. In addition, although ALVE are typically negatively associated with productivity in a commercial setting, their potential role in defence against exogenous ALV may provide an overall net benefit in the productivity of indigenous chickens.

## Supplementary information

**Additional file 1.** All ALVE matrix. This document contains a list of all identified ALVE ordered according to their Galgal5 coordinates, with their name, target site duplication, and previous ambiguous names, where applicable. The genotype of each ALVE is indicated by 0 for homozygous wild type, 0.5 for heterozygotes and 1 for ALVE insertion homozygotes. This file also includes all Ethiopian and Nigerian regional abbreviations.

**Additional file 2: Table S1.** Sampled populations and their identified ALVE diversity. The table includes how many individuals were sampled in each site, the total number of different ALVE identified in those birds, and the number of those which were only found in that region. **Table S2.** Individual chicken samples selected for PCR validation of bioinformatically detected sites by obsERVer. This table includes the 20 randomly selected ALVE to validate the findings of obsERVer, the selected individuals and their bioinformatically-predicted genotype. **Table S3.** Diagnostic ALVE PCR assays designed for obsERVer validation. This table lists the PCR primers for the obsERVer validation and the predicted and product length for each allele. **Table S4.** ALVE distribution relative to coding features and randomly simulated integrations. This table lists the observed genomic distribution of ALVE relative to coding features when compared with a model of random integration. These values support Fig. 3. **Table S5.** ALVE distribution relative to coding feature regions and randomly simulated integrations. This table pairs with Table S4 and shows the observed and simulated values for ALVE integration within exons, UTRs and introns.

**Additional file 3: Figure S1.** Agilent 4200 TapeStation results for 20 diagnostic assays used for the validation of the bioinformatically detected ALVE integrations by obsERVer. PCR results for 20 ALVE detected by obsERVer selected to validate the bioinformatically detected integrations.

**Additional file 4: Figure S2.** Phylogeny of sampled birds on ALVE genotype. Dendrogram of all individuals based on their ALVE content. Figure 2 indicates population structure in a similar manner, but this supplementary figure labels each individual dataset.

## Data Availability

Additional file 1 accompanying this manuscript contains a complete list of the ALVE with their locations and the individuals in which they were identified. The obsERVer pipeline is freely available on GitHub (https://github.com/andrewstephenmason/obsERVer). The Galgal5 reference genome is available on GenBank (GCF_000002315.4). WGS data is available from the authors upon reasonable request. All transfer of samples, data analysis and sharing complies with the principles set out in the Nagoya Protocol.
