# Supplementary material for: Diversity of endogenous avian leukosis virus subgroup E (ALVE) insertions in indigenous chickens
Source: Genet Sel Evol. 2020 Jun 1;52:29. doi: 10.1186/s12711-020-00548-4 (PMC7268647; doi:10.1186/s12711-020-00548-4)
Supplement: Supplementary file 2 — Additional file 2: Table S1. Sampled populations and their identified ALVE diversity. The table includes how many individuals were sampled in each site, the total number of different ALVE identified in those birds, and the number of those which were only found in that region. Table S2. Individual chicken samples selected for PCR validation of bioinformatically detected sites by obsERVer. This table includes the 20 randomly selected ALVE to validate the findings of obsERVer, the selected individuals and their bioinformatically-predicted genotype. Table S3. Diagnostic ALVE PCR assays designed for obsERVer validation. This table lists the PCR primers for the obsERVer validation and the predicted and product length for each allele. Table S4. ALVE distribution relative to coding features and randomly simulated integrations. This table lists the observed genomic distribution of ALVE relative to coding features when compared with a model of random integration. These values support Fig. 3. Table S5. ALVE distribution relative to coding feature regions and randomly simulated integrations. This table pairs with Table S4 and shows the observed and simulated values for ALVE integration within exons, UTRs and introns. [file 12711_2020_548_MOESM2_ESM.docx]

**Table S1. Sampled populations and their identified ALVE diversity.** Regions are split by country, with the abbreviated codes used in the full individual-level ALVE matrix (additional file 1). The table includes how many individuals were sampled in each site, the total number of different ALVEs identified in those birds, and the number of those which were only found in that region. The Iraqi regions were broader than villages, so have been given generic names as well as which samples in the individual-level matrix were within each region.

|  | **Region Name** | **Code** | **Individuals** | **ALVEs** | **ALVEs unique to region** |
| --- | --- | --- | --- | --- | --- |
| **Ethiopia** | Fagita Lekoma Batambie | ABB | 7 | 90 | 17 |
|  | Banja Surta | ABS | 9 | 81 | 11 |
|  | Fagita Lekoma Amesha Shinkuri | AFA | 10 | 53 | 0 |
|  | Dulecha Hugub | AFDH | 10 | 69 | 13 |
|  | Dylecha Kefis | AFDK | 9 | 66 | 13 |
|  | Fagita Lekoma Gafera | AFG | 9 | 63 | 16 |
|  | Gondar Zuria Tsion Teguaz | AGT | 10 | 68 | 7 |
|  | Kalu Adane | AK025A | 10 | 53 | 11 |
|  | Kalu Arabo | AKA | 10 | 49 | 7 |
|  | Menz Gera Midir Alfa Midir | AMAM | 10 | 62 | 8 |
|  | Menz Gera Midir Negasi Amba | AMNA | 10 | 48 | 3 |
|  | South Achefer Ashuda | ASA | 10 | 70 | 11 |
|  | South Achefer Dikuli | ASD | 10 | 60 | 13 |
|  | Dibate Gesses | BGDG | 10 | 53 | 3 |
|  | Dibate Kido | BGDK | 9 | 72 | 8 |
|  | Debre Zeit Horro | DZ | 31 | 91 | 16 |
|  | Dugda Bekele Girissa | ODB | 10 | 71 | 14 |
|  | Dugda Shubi Gemo | ODS | 10 | 51 | 15 |
|  | Dara Kumato | SDK | 10 | 76 | 5 |
|  | Dara Loya | SDL | 10 | 64 | 10 |
|  | Enderta Meseret | TGEN | 10 | 44 | 6 |
|  | Merebleke Hadush Adi | TMLHA | 8 | 53 | 0 |
|  | Merebleke Mihquan | TMLM | 9 | 79 | 18 |
|  | Sahareti Samire Gijet | TSSG | 9 | 80 | 9 |
|  | Sahareti Samire Metkilimat | TSSM | 10 | 81 | 10 |
| **Nigeria** | Dakan-Karu | DK | 10 | 66 | 2 |
|  | Degema | De | 10 | 86 | 2 |
|  | Gitta Mbasha | GM | 10 | 95 | 5 |
|  | Isiokpo | Is | 10 | 72 | 6 |
|  | Jiga | Ji | 4 | 51 | 4 |
|  | Karu | Ka | 10 | 79 | 4 |
|  | Kwendo/Doro/Tadurga | KDT | 6 | 71 | 14 |
|  | Odenkume | Od | 10 | 78 | 2 |
|  | Ogbondoroko | Og | 10 | 88 | 8 |
|  | Okpofe | Ok | 10 | 74 | 1 |
|  | Sabiyal | Sab | 6 | 76 | 9 |
|  | Sanchitagi | San | 9 | 96 | 12 |
|  | Senchi | Sen | 4 | 47 | 5 |
|  | Wat-Karu | WK | 10 | 69 | 2 |
| **Iraq** | Region 1 (95-105) | - | 9 | 67 | 30 |
|  | Region 2 (50-61) | - | 5 | 33 | 13 |
|  | Region 3 (1-14) | - | 13 | 88 | 29 |

**Table S2: Individual chicken samples selected for PCR validation of bioinformatically detected sites by obsERVer.** Names for the twenty randomly selected ALVEs are shown, plus six samples used for the assay following the IDs used in AF1 and explained above in Table S1. Individuals containing the ALVE integration are underlined, with those homozygous for the ALVE highlighted in bold. All three genotypes were represented where possible. Primer sequences for each assay are reported in Table S3.

| **ALVE** | **Selected individuals** |
| --- | --- |
| ALVE_ros117 | AMNA-5C-138, AMNA-7H-144, ASA-4C, ASA-8C, De-1D09, De-1G08 |
| ALVE_ros218 | **AFDH-C7**, AFDH-H2, **AFDH-H6**, AFDH-H8, KDT-1E02, KDT-1F02 |
| ALVE_ros285 | AK025A-10C-059, AK025A-1HC-55, AK025A-2H-149, AK025A-8C, **De-1B09**, Is-1G12 |
| ALVE_ros299 | AFDH-H2, **AFDH-H9**, **AFDK-H9**, AMAM-2C-151, KDT-1E02, KDT-1F02 |
| ALVE_ros453 | AFDK-H6, AFDK-H9, De-1D09, De-1E08, **De-1F09**, **De-1G08** |
| ALVE_ros475 | BGDK-6H, BGDK-7H, BGDK-9H, GM-1C01, Ok-1B11, Ok-1E11 |
| ALVE_ros631 | AFDK-H6, AFDK-H7, AFDK-H9, AK025A-2H-149, AK025A-8C, San-2F03 |
| ALVE_ros642 | BGDK-3C, BGDK-5H-82, BGDK-6H, BGDK-7H, BGDK-9H, **San-2G02** |
| ALVE_ros650 | BGDK-11H-56, BGDK-12C, BGDK-3C, BGDK-5H-82, BGDK-6H, Sen-1F04 |
| ALVE_ros664 | AKA-4H, AMAM-2C-151, **AMNA-5C-138**, **AMNA-7H-144**, ASA-1C, ASA-4C |
| ALVE_ros718 | AFDH-C3, AFDH-C5, AFDH-C7, AFDH-H6, AFDH-H8, **SDL-3C-019** |
| ALVE_ros723 | **BGDK-5H-82**, BGDK-6H, BGDK-7H, **BGDK-9H**, De-1B09, De-1D09 |
| ALVE_ros768 | **BGDK-11H-56**, BGDK-6H, BGDK-7H, KDT-1E02, KDT-1F02, Ok-1B11 |
| ALVE_ros910 | **AFDK-H6**, AK025A-2H-149, BGDK-11H-56, **BGDK-12C**, KDT-1E02, KDT-1F02 |
| ALVE_ros912 | ABS-5H1, ABS-6H, AK025A-10C-059, De-1B09, De-1D09, De-1E08 |
| ALVE_ros916 | AFDH-C3, AFDH-C5, AFDK-H7, De-1B09, De-1D09, De-1E08 |
| ALVE_ros919 | **AGT-9C**, **AKA-4H**, ASA-4C, ASA-8C, De-1B09, De-1D09 |
| ALVE_ros981 | AFDH-C5, AFDH-H6, AFDH-H8, AFDH-H9, AFDK-H6, AFDK-H7 |
| ALVE_ros984 | AFDH-H1, AK025A-10C-059, AK025A-1HC-55, AK025A-2H-149, AK025A-8C, AKA-4H |
| ALVE_ros1001 | AFDH-C3, AFDH-C5, AFDH-C7, BGDK-3C, BGDK-6H, BGDK-7H |

**Table S3: Diagnostic ALVE PCR assays designed for obsERVer validation.** All assays follow a three-primer approach. The first and second primers in each set are unique forward and reverse primers respectively, which anneal to the flanking genomic DNA. The third is an ALVE “alternative primer”, which anneals to the integrated sequence, if present. High sequence integrity in the LTRs facilitated reuse of the alternative primers across multiple assays.

| **ALVE** | **Primers** | **ALVE + (bp)** | **ALVE – (bp)** |
| --- | --- | --- | --- |
| ALVE_ros117 | 5'-TCTTGTGAAGGTGCTGCTGT  5'-GCATGGACTGTGGGGAATGA  5'-GTGCACCTGGGTAGATGGAC (ALVE-alt1) | 260 | 360 |
| ALVE_ros218 | 5'-CGCAACATCGATTGGCATGA  5'-CACAAGTAGGTCAGGTGCCC  5'-GTGCACCTGGGTAGATGGAC (ALVE-alt1) | 160 | 310 |
| ALVE_ros285 | 5'-CCACTCCCTGCTGTCACTTT  5'-AACTTGCTCCTTCCCTTGCA  5'-GTGCACCTGGGTAGATGGAC (ALVE-alt1) | 178 | 375 |
| ALVE_ros299 | 5'-AGCAGTTACCAGGAGTCGAA  5'-TGGAAACTGTGCTGGCTTCT  5'-CTAGTCGCCACCAATGAGCA (ALVE-alt2) | 282 | 352 |
| ALVE_ros453 | 5'-GATCACCTCCAACACATGCTG  5'-TTTCCCACTGCTGAAGGACC  5'-GTGCACCTGGGTAGATGGAC (ALVE-alt1) | 288 | 400 |
| ALVE_ros475 | 5'-AAGTTGCAGCAAAGTTGGC  5'-GGTGTCACAGTGGTAGCCTC  5'-GATTGCGAACACCTGAATGAAG (ALVE-alt3) | 126 | 338 |
| ALVE_ros631 | 5'-CGTTCTGCTGGTGTTCTTTGG  5'-ACAGTTAACGTGTGTCTGGGT  5'-CTAGTCGCCACCAATGAGCA (ALVE-alt2) | 290 | 402 |
| ALVE_ros642 | 5'-CGAGAAATCCAGCTCTTAGGGT  5'-ACGGCTTCCTTTCTTTTGGT  5'-GTGCACCTGGGTAGATGGAC (ALVE-alt1) | 300 | 390 |
| ALVE_ros650 | 5'-TCGCTCAAATGAAGGGCAAA  5'-AGCCAGAAACGCAGAACAGA  5'-CTAGTCGCCACCAATGAGCA (ALVE-alt2) | 214 | 338 |
| ALVE_ros664 | 5'-CGAGGGTTGATGTGATGGGT  5'-AGAGCTCTGGATTGGACCCA  5'-GTGCACCTGGGTAGATGGAC (ALVE-alt1) | 178 | 255 |
| ALVE_ros718 | 5'-TCTCTTAGCTCCATCCACTCA  5'-TGCAGTTACCTAGGATGGCAG  5'-CTAGTCGCCACCAATGAGCA (ALVE-alt2) | 372 | 435 |
| ALVE_ros723 | 5'-TCCATCCCCAACACTTACCC  5'-TGACTTCACCAGAGCAAAGCA  5'-GTGCACCTGGGTAGATGGAC (ALVE-alt1) | 305 | 365 |
| ALVE_ros768 | 5'-TAGGCAGGCTGAGAGGTTCA  5'-ACCACAATGCTTTTAGGCCAG  5'-GTGCACCTGGGTAGATGGAC (ALVE-alt1) | 226 | 362 |
| ALVE_ros910 | 5'-AGGGTGTTAGTCTGAAGCACG  5'-CGACACAGTGTTGCAAGCAA  5'-CTAGTCGCCACCAATGAGCA (ALVE-alt2) | 174 | 304 |
| ALVE_ros912 | 5'-GGTTATGTGCTACCCCTGAAGT  5'-AGAGTGAAAGGGCTTGCCAA  5'-GTGCACCTGGGTAGATGGAC (ALVE-alt1) | 231 | 358 |
| ALVE_ros916 | 5'-TGGCTACGCTGGGAATTGAA  5'-GTCGATTTACACCAGCTCAGGA  5'-GTGCACCTGGGTAGATGGAC (ALVE-alt1) | 281 | 434 |
| ALVE_ros919 | 5'-GCGTGCAGTAAGAATGTCAGC  5'-CAGCATGTTCGTCCAGACAT  5'-GTGCACCTGGGTAGATGGAC (ALVE-alt1) | 184 | 352 |
| ALVE_ros981 | 5'-AGCAGTGAGAAGCAGCAAGT  5'-TCAAAGTAACTGCAGGGGACA  5'-CTAGTCGCCACCAATGAGCA (ALVE-alt2) | 209 | 347 |
| ALVE_ros984 | 5'-TTGGCTGTCTTCATCTCTGAC  5'-CTTTCCAGTCTAAGTGCAAATCA  5'-CTAGTCGCCACCAATGAGCA (ALVE-alt2) | 207 | 382 |
| ALVE_ros1001 | 5'-TCGTTACCCAGTGCCAATCC  5'-AAGTGGTGTGGACAGAAGCT  5'-GTGCACCTGGGTAGATGGAC (ALVE-alt1) | 278 | 350 |

**Table S4: ALVE distribution relative to coding features and randomly simulated integrations.** These values match Figure 3 in the manuscript. Simulated values represent the average percentage in each category from one million randomly simulated redistributions of an equal number of integrations (n=974). Standard deviations of the simulation are also shown. Categories represent either overlap with a coding region (CR) or the 10kbp bin up to the indicated distance. Distances derived using the shortest distance to the nearest Galgal5 Ensembl v87 gene build feature.

| **Category** | **Observed (%)** | **Simulated (%)** | **Simulated SD** | **Binomial Test P value** |
| --- | --- | --- | --- | --- |
| CR | 40.7 | 51.8 | 3.4 | 1.74 x10^-14^ |
| 10k | 17.5 | 4.1 | 0.1 | 7.16 x10^-19^ |
| 20k | 4.9 | 5.3 | 0.9 | 0.567 |
| 30k | 4.3 | 4.2 | 0.2 | 0.936 |
| 40k | 3.9 | 4.1 | 0.6 | 0.746 |
| 50k | 3.5 | 3.7 | 0.4 | 0.799 |
| >50k | 25.2 | 26.8 | 2.1 | 0.262 |

**Table S5: ALVE distribution relative to coding feature regions and randomly simulated integrations.** These data are derived from the coding region values in Figure 3 and Table S2 and show the overlaps within coding regions divided into exons, UTRs and introns.

| **Category** | **Observed (%)** | **Simulated (%)** | **Simulated SD** | **Binomial Test P value** |
| --- | --- | --- | --- | --- |
| Exon | 1.5 | 4.9 | 0.7 | 6.36 x10^-4^ |
| UTR | 0.8 | 3.1 | 0.3 | 3.30 x10^-3^ |
| Intron | 97.7 | 92.0 | 4.1 | 1.78 x10^-6^ |
