# Supplementary figures and images for: Diversity of endogenous avian leukosis virus subgroup E (ALVE) insertions in indigenous chickens
Source: Genet Sel Evol. 2020 Jun 1;52:29. doi: 10.1186/s12711-020-00548-4 (PMC7268647; doi:10.1186/s12711-020-00548-4)

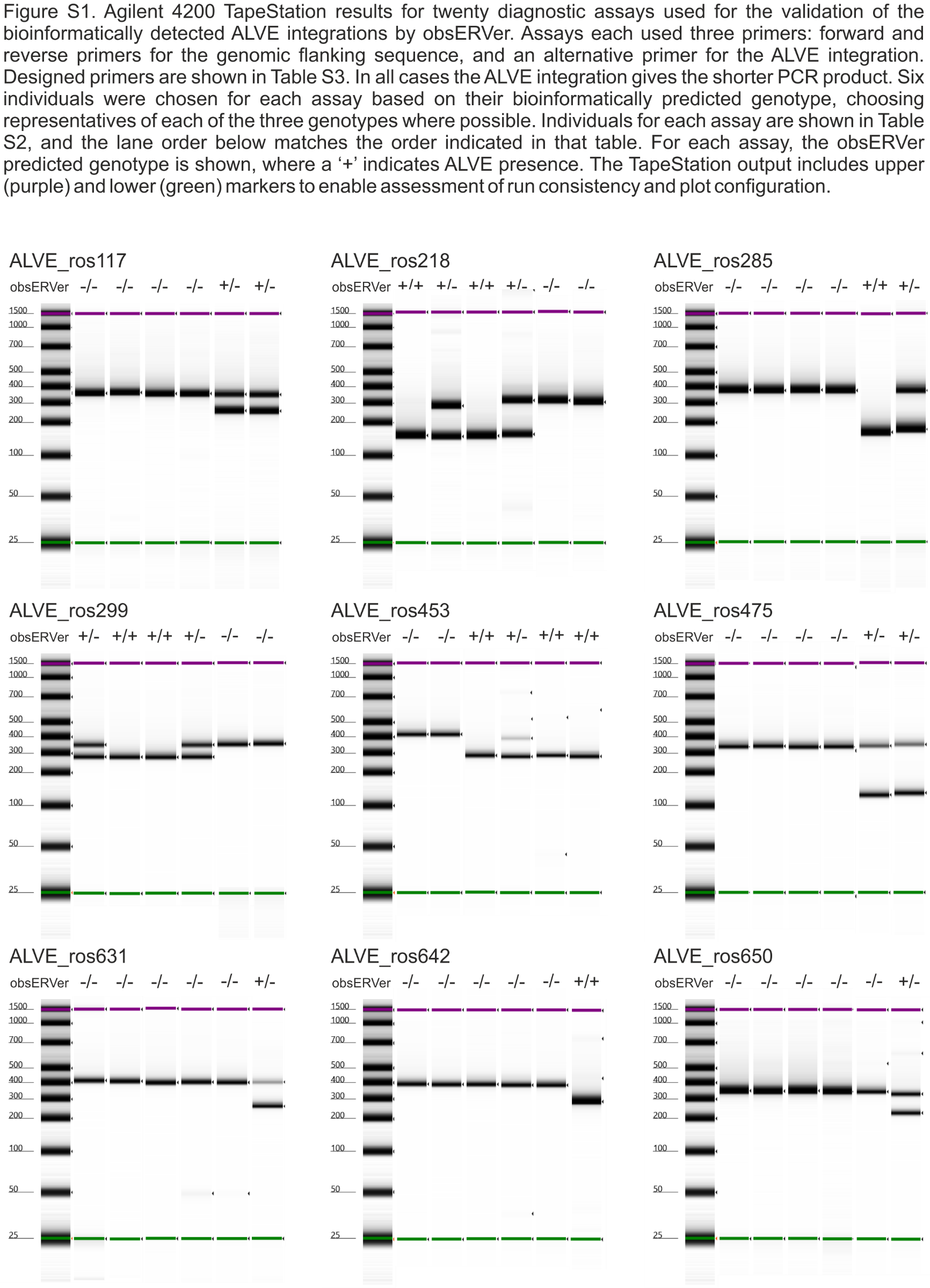


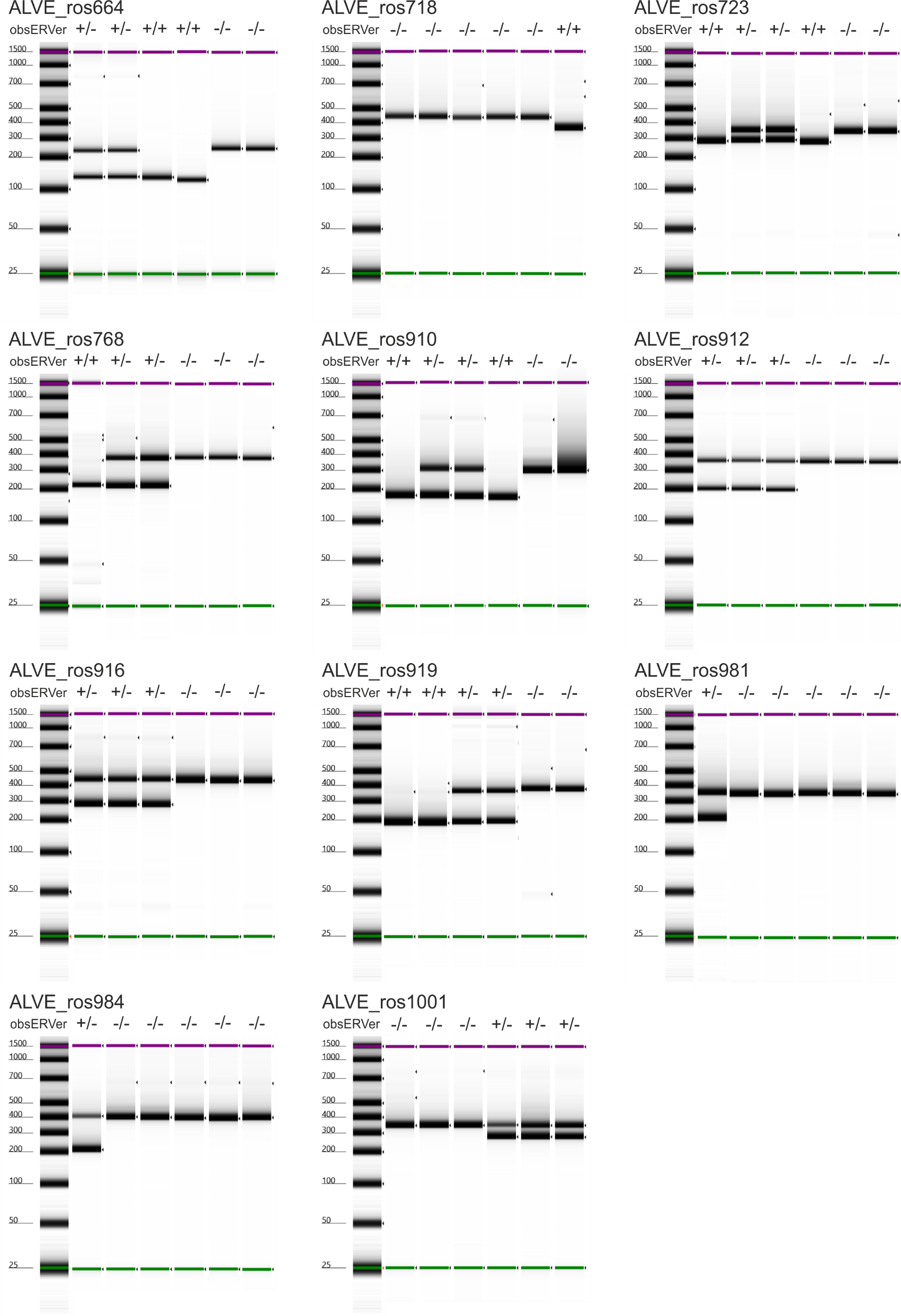

Supplement: Supplementary file 3 — Additional file 3: Figure S1. Agilent 4200 TapeStation results for 20 diagnostic assays used for the validation of the bioinformatically detected ALVE integrations by obsERVer. PCR results for 20 ALVE detected by obsERVer selected to validate the bioinformatically detected integrations. [file 12711_2020_548_MOESM3_ESM.docx]
