# Supplementary material for: Diversity of endogenous avian leukosis virus subgroup E (ALVE) insertions in indigenous chickens
Source: Genet Sel Evol. 2020 Jun 1;52:29. doi: 10.1186/s12711-020-00548-4 (PMC7268647; doi:10.1186/s12711-020-00548-4)

Figure S2. Phylogeny of sampled birds on ALVE genotype. Branches have been coloured by country to match Figure2: Ethiopia in black, Iraq in red, Nigeria in blue. Individual samples are labelled with regional codes as detailed in AF1 and the individual sequencing ID. Nigerian samples do not cluster by regional geography, but many Ethiopian samples do. The Iraqi samples cluster within the Ethiopian clade, as suggested by Figure 2. Dendrogram constructed with average linkage using the Jaccard distance metric.

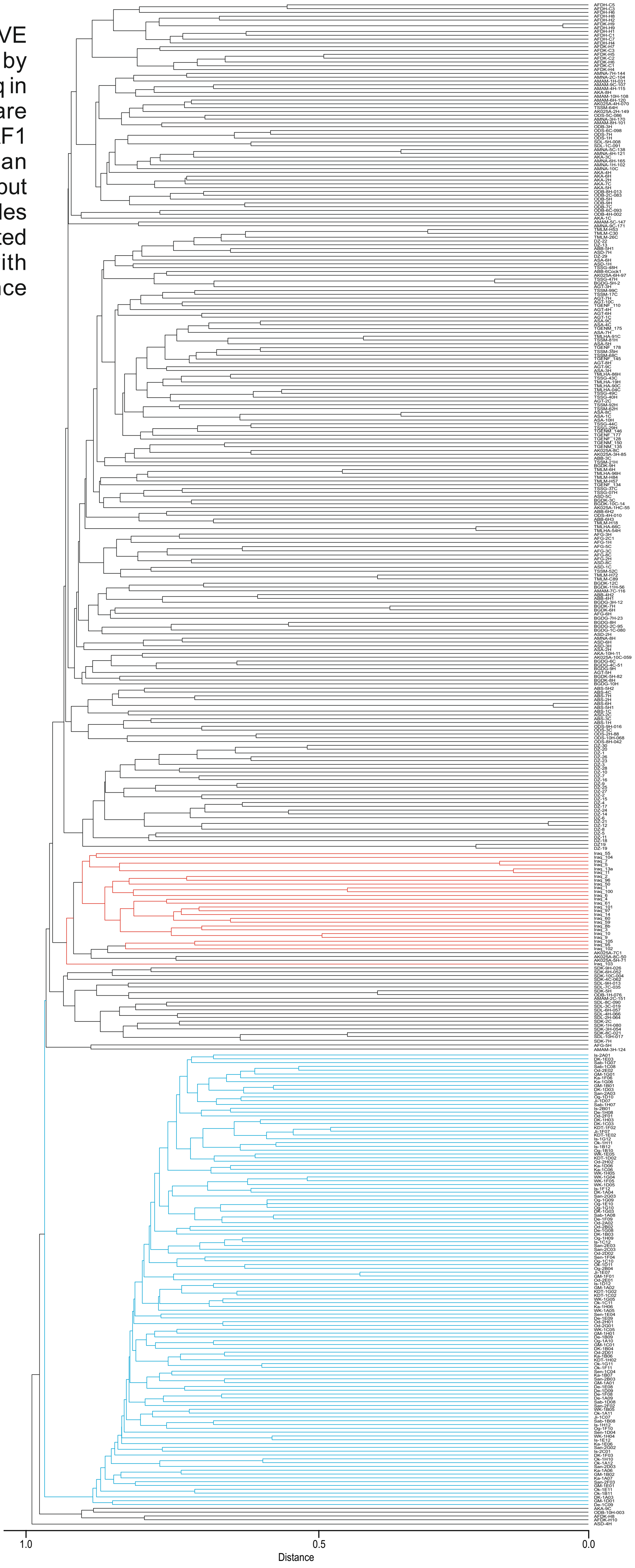

Supplement: Supplementary file 4 — Additional file 4: Figure S2. Phylogeny of sampled birds on ALVE genotype. Dendrogram of all individuals based on their ALVE content. Figure 2 indicates population structure in a similar manner, but this supplementary figure labels each individual dataset. [file 12711_2020_548_MOESM4_ESM.pdf]
